# [Retracted] Huanglian-Houpo drug combination ameliorates H1N1-induced mouse pneumonia via cytokines, antioxidant factors and TLR/MyD88/NF-κB signaling pathways

**DOI:** 10.3892/etm.2025.12931

**Published:** 2025-07-28

**Authors:** Li Zhang, Bei Zhang, Linjing Wang, Mingwu Lou, Yunxia Shen

Exp Ther Med 21:428, 2021; DOI: 10.3892/etm.2021.9845

Following the publication of the above paper, it was drawn to the Editor’s attention by a pair of the listed authors, Mingwu Lou and Yunxia Shen of the Department of Radiology, Shenzhen Clinical Medical School, Guangzhou University of Chinese Medicine, who also were listed on the paper as the corresponding authors, that they were unaware of the submission of this paper to the Journal *Experimental and Therapeutic Biology*.

In view of the fact that all authors must agree to the submission of an article, and also to have read and approved of the final version of the manuscript, and this situation clearly could not have applied in this case, the Editor of *Experimental and Therapeutic Medicine* has decided that this paper should be retracted from the journal. The Editor apologizes to the readership for any inconvenience caused.

